# Investigating Reactive Astrocytosis in Alzheimer's Disease Using [18F]Fluorodeprenyl‐D2 PET Imaging

**DOI:** 10.1002/alz.092151

**Published:** 2025-01-09

**Authors:** Boris‐Stephan Rauchmann, Selim Üstün Guersel, Carolin Isabella Kurz, Simon Lindner, Nicolai Franzmeier, Katharina Buerger, Johannes Gnörich, Mirlind Zaganjori, Letitia Vogler, Laura Sanzo, Klaus Seelos, Robert Perneczky, Matthias Brendel

**Affiliations:** ^1^ LMU University Hospital, Munich Germany; ^2^ Institute for Stroke and Dementia Research, Ludwig‐Maximilians‐Universität München, LMU München, Munich Germany; ^3^ Institute for Stroke and Dementia Research (ISD), University Hospital, LMU, Munich Germany

## Abstract

**Background:**

Alzheimer's Disease (AD) is often accompanied by neuroinflammation, which manifests prior to significant cognitive decline. Reactive astrocytosis is a hallmark of such inflammation, potentially serving as an early biomarker for AD pathology. Our study employs [18F]fluorodeprenyl‐D2 ([18F]F‐DED) positron emission tomography (PET) imaging to in vivo quantify astrocytosis comparing AD with healthy controls and examines its assocciation with cognitive deterioration in AD.

**Method:**

Our cross‐sectional study involved 12 early‐stage Alzheimer's Disease (AD) patients and 11 healthy controls, all undergoing [18F]F‐DED and MRI imaging. AD diagnosis was based on CSF amyloid‐β42/40 ratios under 5.5% and MMSE scores below 27, while controls had MMSE scores above 27 with no amyloid‐β pathology. We assessed differences in [18F]F‐DED uptake using ROI‐based analyses, processing data in PMOD software. This involved calculating the radioligand's volume of distribution in tissues and image‐derived plasma at equilibrium. For ROI analysis, we adapted the Automated Anatomical Labeling (AAL) atlas to each participant's gray matter masks and [18F]F‐DED maps, computing median uptake values per ROI. Cortical amyloid deposition was measured using [18F]flutemetamol β‐amyloid‐PET imaging. CSF biomarkers, including Aβ42, Aβ40, and phosphorylated tau, were analyzed using the Lumipulse G1200 platform. Cognitive performance was evaluated with the MMSE. We used ANCOVA and linear regression in R to explore correlations between cognitive performance and [18F]F‐DED uptake, adjusting for age and sex.

**Results:**

Increased [18F]F‐DED uptake was observed in AD‐affected areas comparing AD with controls, particularly in the bilateral occipital (Left: T:2.28, p=0.03; Right: T:2.51, p=0.02), temporal (Left: T:2.21, p=0.03; Right: T‐Stat 2.22, p=0.03), and frontal lobes (Left: T:2.46, p<0.02; Right: T:2.45, p<0.02). This increased uptake was significantly correlated with cognitive decline, especially in temporal regions, associated with lower cerebrospinal fluid amyloid‐β ratios and reduced MMSE scores (p<0.01), consistent after adjusting for age and gender.

**Conclusion:**

Our findings indicate that [18F]F‐DED PET imaging can detect reactive astrocytosis in AD‐sensitive areas, potentially preceding severe cognitive symptoms. The correlation between increased tracer uptake and cognitive impairment underscores the value of [18F]F‐DED as an AD progression biomarker, offering promising prospects for early diagnosis and intervention in AD's initial stages.

